# Reply to: Triplet-triplet annihilation in rubrene/C60 OLEDs with electroluminescence turn-on breaking the thermodynamic limit

**DOI:** 10.1038/s41467-019-12598-4

**Published:** 2019-10-15

**Authors:** Sebastian Engmann, Adam J. Barito, Emily G. Bittle, Noel C. Giebink, Lee J. Richter, David J. Gundlach

**Affiliations:** 1 Theiss Research, La Jolla, CA 92037 USA; 2 Nanoscale Device Characterization Division, National Institute of Standards and Technology, 100 Bureau Drive, Gaithersburg, MD 20899 USA; 3 Department of Electrical Engineering, The Pennsylvania State University, Electrical Engineering West, State College, PA, 16801 USA; 4 Materials Science and Engineering Division, National Institute of Standards and Technology, 100 Bureau Drive, Gaithersburg, MD 20899 USA

**Keywords:** Lasers, LEDs and light sources, Physics

**Replying to** X. Qiao & D. Ma. *Nature Communications* 10.1038/s41467-019-12597-5 (2019)

In ref. ^1^, we reported a detailed analysis of the electroluminescence (EL) characteristics for a series of rubrene/C_60_ organic light emitting diodes (OLEDs) that exhibit low voltage threshold for detectable emission. Based on extensive kinetic models, we conclude that nonlinear processes are not necessary to describe the emission of the baseline device but can be significant under certain interlayer modifications. In ref. ^2^, Qiao and Ma question our recent analysis of these devices by pointing out that the rate of photon emission exceeds the limit set by the generalized Planck (GP) equation reproduced for reference below:

1 $${\mathrm{d}}N = \frac{{2a\left( \lambda \right)c}}{{\lambda ^4}}\frac{1}{{\exp \left( {\frac{{\frac{{hc}}{\lambda } \,-\, eV}}{{k_{\mathrm{{B}}}T}}} \right) - 1}}{\mathrm{d}}\lambda,$$

with *a* being the absorptivity of the material, *h* the Planck constant, *λ* the wavelength, *k*_B_ the Boltzmann constant, *c* the speed of light, and *T* the temperature of the lattice. Eq. (1) is known to hold for the EL of crystalline and amorphous inorganic semiconductors^3, 4^, where it quantitatively predicts both the form and magnitude of the EL spectrum. This is not the case for the analysis in ref. ^2^, where imposing a sharp bandgap cutoff at ~2.2 eV for rubrene formally precludes any emission below this energy according to Eq. (1), in sharp contrast to the observed rubrene EL spectrum in Fig. 1. This large spectral discrepancy in applying Eq. (1) naturally questions the validity of its use in predicting the associated EL intensity.

**Fig. 1:**
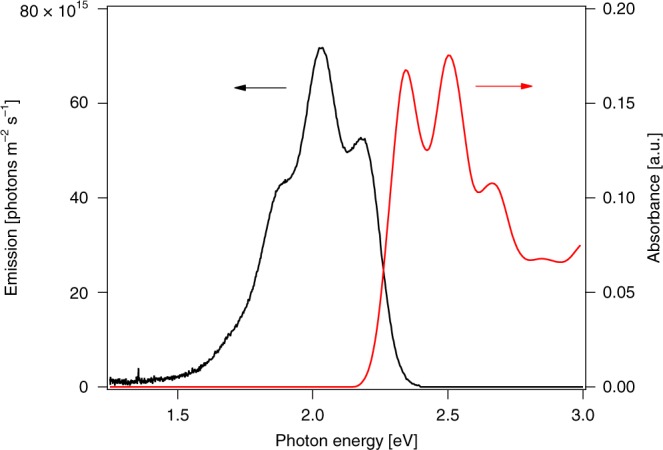
EL spectrum for a rubrene/C_60_ heterojunction as reported in the original manuscript for an applied voltage of 1.15 V. The emission at this level is 3 cd m^−2^. Also shown is the absorbance of a 40-nm-thick film of rubrene

To this point, Eq. (1) was derived from a generalized Kirchhoff relationship under the assumption of chemical equilibrium between the photons and the electron-hole (e-h) gas. Therefore, violation of Eq. (1) is a violation of the assumption of chemical equilibrium. As pointed out in the derivation of Eq. (1) by Würfel^4^, chemical equilibrium between the photons and thee-h gas requires that the rates of photon emission and absorption are fast compared to the driving rate (carrier injection), a condition assumed to be met by systems with absorption ≈1. Würfel explicitly derives Eq. (1) for the case of a long diode (1/*α* » length), and demonstrates its applicability in thick inorganic devices. Most solar cell devices are in this limit. Shown in Fig. 1 is the EL spectrum of an unmodified rubrene/C_60_ device from ref. ^1^, along with the rubrene absorption, estimated from the film thickness and the dielectric function from ellipsometry. Two relevant aspects of the devices are apparent. The first is that the films are optically thin (with *a* < 0.2) and the second is the Stokes shift of the EL. Thus, we see that there are two obvious places where our device can deviate from the assumption of chemical equilibrium: breakdown of detailed balance between the photons and emissive singlets due to weak absorption or breakdown of detailed balance between emissive singlets and the e-h gas.

Qiao and Ma suggest a specific mechanism to account for the deviation from chemical equilibrium observed in our devices: triplet–triplet annihilation (TTA). Their hypothesis can be framed as: assume triplets are in equilibrium with the charge transfer (CT) states, which are in equilibrium with the e-h gas with chemical potential *μ*_T/CT_ = *qV*_a_ and singlets are in equilibrium with triplets with chemical potential *μ*_S_ = 2*μ*_T_, then *μ*_S_ = 2*qV*_a_. This generates a singlet population that clearly is not in direct equilibrium with the e-h gas and implies greater than GP emission. However, this results in *μ*_S_ > *hν* corresponding to negative entropy in the photon field. As pointed out by Würfel, the chemical potential of the photons must be less than the lowest photon energy in the photon bath considered and the lowest detectable emission energy in Fig. 1 is ~1.6 eV. Therefore, a quantitative treatment of the chemical potential in a TTA model requires a detailed kinetic analysis, as we performed in ref. ^1^. The TTA hypothesis is not a unique means to deviate from chemical equilibrium. As noted above, the photon field need not obey detailed balance with the material system at all. Additionally, singlet-to-carrier reverse reactions (i.e. dissociation of rubrene singlet excitons at the donor–acceptor interface) can result in anomalous singlet populations as is found in the operation of a 3-level laser. Similarly, retention of the exothermicity of the Stokes shift in the emissive states (hot excitons) will result in failure of the chemical equilibrium hypothesis^5, 6^. Simple estimates of the luminance predicted by Eq. (1), based on a van Roosbroeck–Shockley^7^ estimate of the singlet absorption, indicate that the assumption of chemical equilibrium between photons and emissive singlets require either a singlet chemical potential of ≈1.6 eV (0.45 eV greater than the applied potential) or a singlet temperature ≈500 K (1.7× room temperature). The latter effectively corresponds to an exponential Urbach absorption tail with a characteristic slope of ~43 meV, comparable to what is observed in many organic semiconductors^8^.

It is critical to emphasize that, given the clear violation of chemical equilibrium in the device, any quantitative treatment of the operation requires a kinetic analysis of the system as that is the only way to calculate the relevant steady-state populations. This was done in ref. ^1^, with the conclusion that TTA did not significantly contribute to emission in the reference device (but did in bathocuproine (BCP) interlayer modified devices). Qiao and Ma do not present a kinetic model, consistent with our devices, requiring TTA. Here we recap the phenomenology reported in ref. ^1^, consistent with our kinetic model, that lead us to conclude that TTA was not required to account for the low threshold emission.

First is our observation that the EL increases when we add a BCP interlayer. Because rubrene triplets are thought to be a product of rubrene/C_60_ CT states^9^, decreasing the CT-state binding energy and formation and dissociation kinetics by adding BCP layer should decrease the triplet density and thus reduce TTA-assisted rubrene emission, opposite to the trend we observe.

Second is the linear functional dependence of *L* vs. *J* that we observe (or equivalently, equal current and EL ideality factors) near the turn-on voltage. For TTA up-conversion to be the dominant source of light emission in this region, *L* inherently depends quadratically on *J* at low current density and only transitions to a linear dependence above a critical triplet density, *T*_trans_ > (*k*_TTA_*τ*)^−1^. Assuming an ideal case where every injected e− and h+ form a triplet, this transition takes place at a current density

2 $$J_{{\mathrm{trans}}} = \frac{{qa_0}}{{k_{{\mathrm{TTA}}}\tau ^2}}.$$

Based on the known values of *k*_TTA_*~*10^−14^ cm^3^ s^−1^ and *τ* = 100 µs measured for rubrene single crystals (which reflect upper bounds to the actual *k*_TTA_ and *τ* expected in disordered thin films such as ours) and the *a*_0_ = 40 nm rubrene thickness in our OLEDs, the ideal minimum threshold current amounts to ~6 mA cm^−2^. This is two orders of magnitude above the current range where we observe linear *L* vs. *J* and thus TTA cannot explain our data.

We thank Qiao and Ma for pointing out that, while minority carrier recombination will always lead to photon emission at arbitrarily low bias voltage, and thus half-gap emission is not intrinsically unusual, a second useful limit is given by the GP equation. However, when GP is violated, the fundamental learning is that the photons are not in chemical equilibrium with the e-h gas. This mandates a detailed kinetic analysis, which was performed in ref. ^1^.

## Data Availability

The data that support the findings of this study are available from the corresponding author upon reasonable request.
